# Bis(μ-thio­semicarbazide-κ^3^*N*^1^,*S*:*S*;κ^3^*S*:*N*^1^,*S*)bis­[(di­methyl­formamide-κ*O*)(thio­semicarbazide-κ^2^*N*^1^,*S*)cadmium(II)] tetra­kis­(2,4,6-tri­nitro­phen­olate): synthesis, crystal structure and Hirshfeld surface analysis

**DOI:** 10.1107/S2056989025003974

**Published:** 2025-05-13

**Authors:** R. Santhakumari, K. Ramamurthi, Manpreet Kaur, Jerry P. Jasinski, Suresh Sagadevan, Helen Stoeckli-Evans

**Affiliations:** a PG & Research Department of Physics, Kalaignar Karunanidhi Government Arts College for Women (Autonomous), Kalaignar Karunanidhi Government Arts College for Women (Autonomous), Pudukkottai-622001, Tamilnadu, India; b Former Professor, Crystal Growth and Thin Film Laboratory, Bharathidasan University, Tiruchirappalli-620024, Tamilnadu, India; c Department of Chemistry Keene State College, 229 Main Street Keene New Hampshire 03435-2001 USA; d Nanotechnology and Catalysis Research Centre, Universiti Malaya, Kuala Lumpur-50603, Malaysia; e Institute of Physics, University of Neuchâtel, Rue Emile-Argand 11, CH-2000, Neuchâtel, Switzerland; University of Aberdeen, United Kingdom

**Keywords:** crystal structure, cadmium complex, thio­semicarbazide, picrate, hydrogen bonding, Hirshfeld surface, fingerprint plots

## Abstract

In the title complex salt, [Cd_2_(C_3_H_7_NO)_2_(CH_5_N_3_S)_4_](C_6_H_2_N_3_O_7_)_4_, (**I**), the binuclear cation is located about a crystallographic center of symmetry. The asymmetric unit of the complex cation is composed of two bidentate thio­semicarbazide ligands and one mol­ecule of di­methyl­formamide coordinated to a cadmium(II) atom. The S atom of one of the thio­semicarbazide ligands bridges the cadmium atoms about the inversion center. The positive charge of the complex is balanced by picrate anions. In the crystal, the cation is linked to the picrate anions by side-by-side bifurcated N—H⋯(O,O) hydrogen bonds in which the central O atom acts as a double acceptor for two such bonds, enclosing *R*^1^_2_(6) and *R*^2^_1_(6) ring motifs. In the crystal, further N—H⋯O hydrogen bonds link the various units to form slabs lying parallel to the (001) plane and the slabs are linked by C—H⋯O hydrogen bonds, thereby forming a three-dimensional network.

## Abstract 2

The complete binuclear cation of the title complex salt is generated by a crystallographic center of symmetry and features bridging S atoms.

## 1. Chemical context

Organic mol­ecules containing π-electron conjugated systems, asymmetrized by the electron donor and acceptor groups, are highly polarizable entities for non-linear optical (NLO) applications (Long, 1995 ▸; Verbiest *et al.*, 1997 ▸; Pal *et al.*, 2004 ▸). Furthermore, in metal–organic complexes the metal-to-ligand bonding is expected to display a large mol­ecular hyper–polarizability due to the transfer of electron density between the metal atom and the conjugated ligand system (McArdle *et al.*, 1974 ▸; Arivanandhan *et al.*, 2005 ▸). A key factor is that the diversity of the central metal, its oxidation state and the ligands make it possible to optimize the charge-transfer inter­actions. In the case of metal–organic coordination complexes, group 12 (group IIB) metals are extensively chosen, as their complexes usually achieve high transparency in the UV region because of their closed *d*^10^ shell (Sun *et al.*, 2003 ▸; Ushasree *et al.*, 1999 ▸), hence *d*–*d* electronic transitions are not possible.

Picric acid (2,4,6-tri­nitro­phenol) is an electron-acceptor forming charge-transfer mol­ecular complexes with a number of electron donor compounds, such as amines, through electrostatic or hydrogen-bonding inter­actions (Anitha *et al.*, 2005 ▸; Saminathan *et al.*, 2005 ▸; Muthamizhchelvan *et al.*, 2005 ▸; Muthu & Meenakshisundaram, 2012 ▸). As a result of the formation of the conjugated base on proton loss to form picrate anions, the magnitude of the mol­ecular hyperpolarizability is increased (Anandha Babu *et al.*, 2010 ▸). Picric acid forms salts with amino acids, such as l-valine and l-asparagine (Anitha *et al.*, 2004 ▸; Braga *et al.*, 2004 ▸). Hydrogen bonds play an important role in the supra­molecular packing and in the generation of non-centrosymmetric structures (Berkovitch-Yellin & Leiserowitz, 1984 ▸; Min Jin *et al.*, 2003 ▸; Frankenbach & Etter, 1992 ▸; Etter & Huang, 1992 ▸; Sherwood, 1998 ▸). Hence, thio­semicarbazide (CH_5_N_3_S) is an inter­esting candidate, as it binds well to most transition metals of groups 7–10. The crystal structure of thio­semicarbazidium picrate monohydrate has been reported (Xie, 2007 ▸) in which extensive hydrogen bonding lead to the formation of a three-dimensional supra­molecular structure.
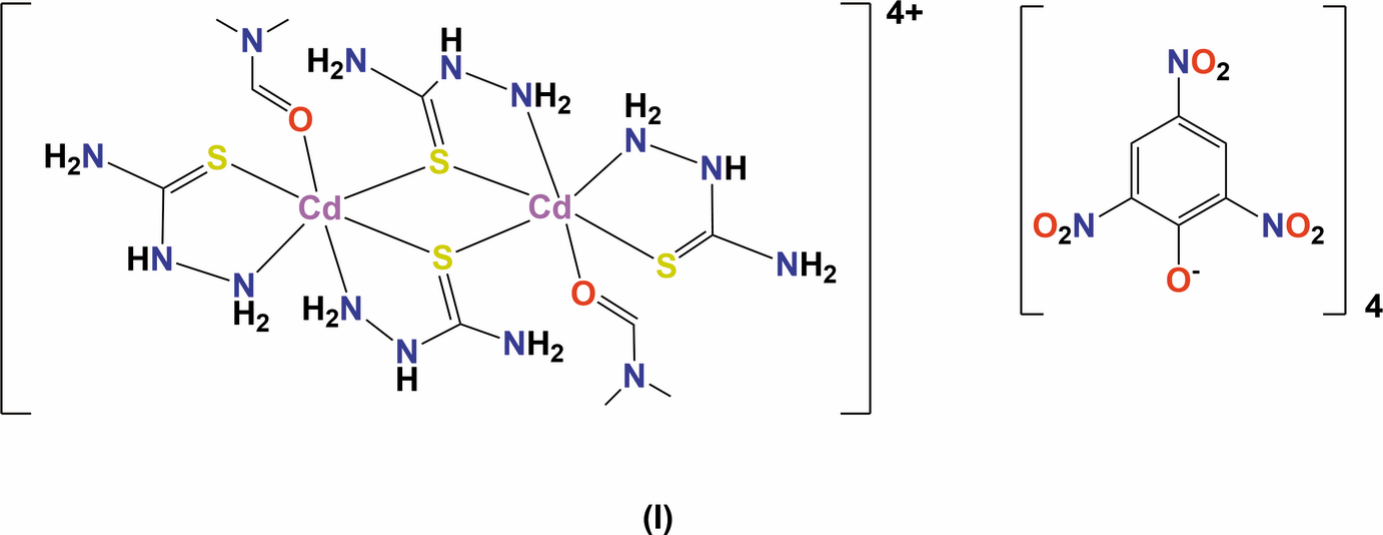

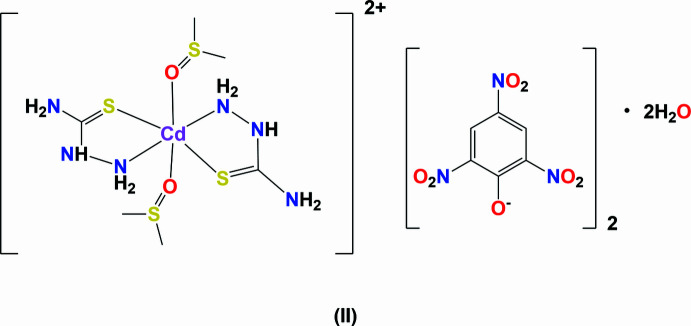


The title compound (**I**), a cadmium thio­semicarbazide picrate, was prepared using di­methyl­formamide as solvent. A search of the Cambridge Structural Database (CSD; V5.46, last update February 2025; Groom *et al.*, 2016 ▸) for cadmium–thio­semicarbazide complexes gave 15 hits. Only one compound involves picrate as anion, namely *trans*-bis­(dimethyl sulfoxide-κ*O*)bis­(thio­semicarbazide-κ^2^*N*^1^,*S*)cadmium bis­(2,4,6-tri­nitro­phenolate) dihydrate (**II**) (CSD refcode QAJDOW; Shanthakumari *et al.*, 2011 ▸), which was prepared using dimethyl sulfoxide (DMSO) as solvent. In both cases the solvent mol­ecule coordinates to the cadmium(II) atom *via* its O atom. Herein, the structures and Hirshfeld surfaces of compounds (**I**) and (**II**) are compared.

## 2. Structural commentary

**Figure 1:**
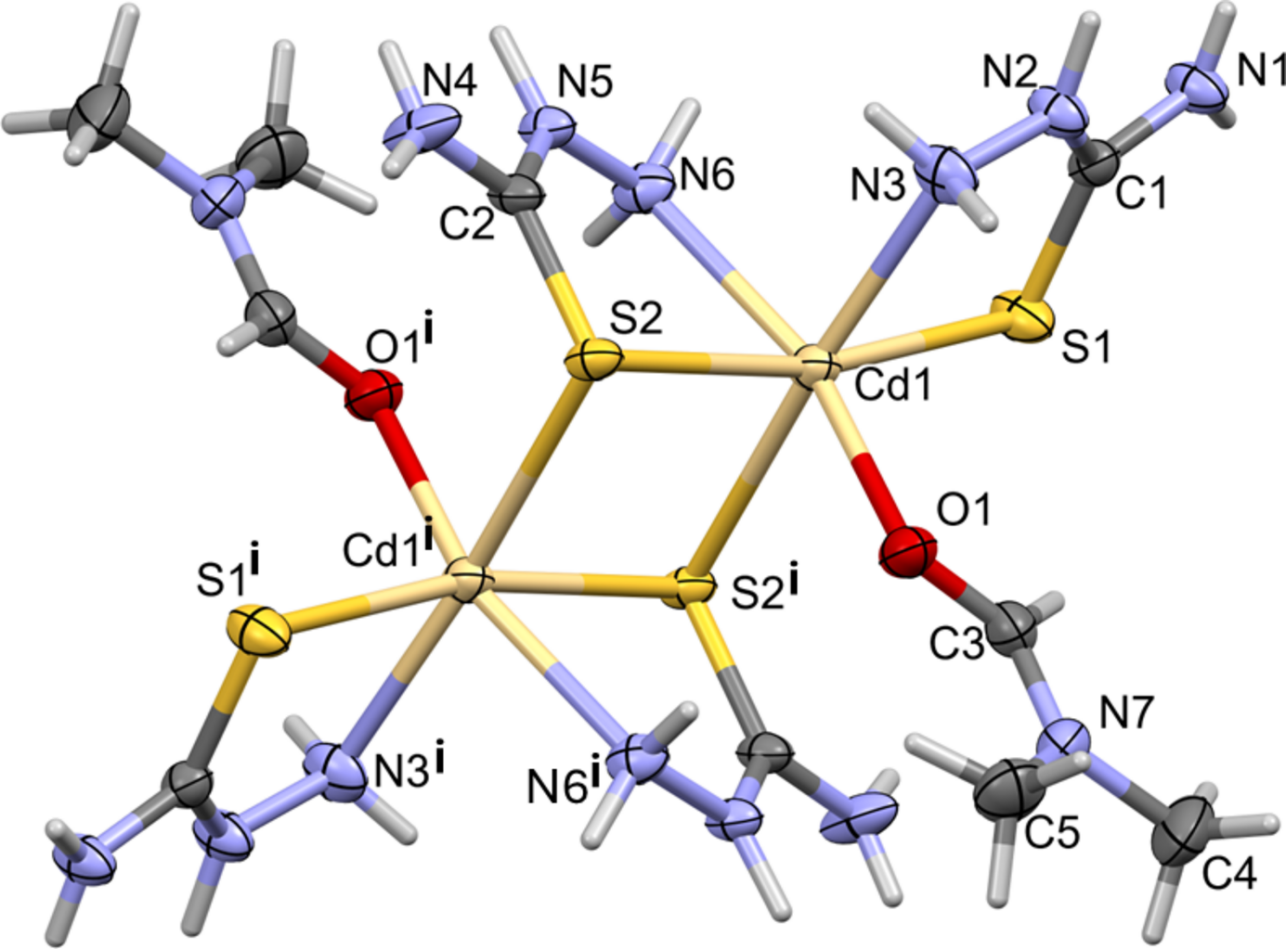
The mol­ecular structure of the complex cation of compound (**I**), with displacement ellipsoids drawn at the 50% probability level. [Symmetry code (i): −*x* + 1, −*y* + 2, −*z* + 1.]

The title complex (**I**), is composed of a [Cd_2_(thio­semicarb­azide)_4_(dimethyl formamide)_2_]^4+^ cation, located about an inversion center, and two crystallographically distinct picrate (2,4,6-tri­nitro­phenolate) anions. The cadmium atom coordinates to atom O1 of a DMF mol­ecule, and to the sulfur (S1 and S2) and nitro­gen (N3 and N6) atoms of two bidentate thio­semicarbazide ligands (Fig. 1 ▸). Atom S2 bridges the cadmium atoms about the inversion center. Selected bond lengths and bond angles for complexes (**I**) and (**II**) are listed in Table 1 ▸.

In the cation of (**I**), atom Cd1 is sixfold coordinated, CdS_2_O_2_N_2_, in a distorted octa­hedral geometry. The structural index, τ_6_, describing the deformation of an octa­hedral coordination sphere has a value of [540° – (160.89 + 167.97° + 171.43°)]/180° = 0.22 for Cd1 (τ_6_ = 0 for a perfect octa­hedral geometry; 0.75 for a trigonal prismatic geometry, and = 1 for a penta­gonal pyramidal geometry; Stoeckli-Evans *et al.*, 2025 ▸). In complex (**II**), the Cd atom is located on an inversion center and is coordinated to the O atom of two DMSO mol­ecules, and to the N and S atoms of two bidentate thio­semicarbazide ligands. The structural index, τ_6_, of the sixfold coordination sphere of the cadmium atom (CdS_2_O_2_N_2_) is [540° – (3 × 180°)]/180° = 0.

In (**I**) the cadmium–nitro­gen bond lengths, Cd1—N3 and Cd1—N6, are similar and close to the value observed for complex (**II**), *viz*., 2.398 (3) and 2.421 (3) Å, respectively in (**I**) compared to 2.382 (2) Å in (**II**). The equivalent Cd—S bond length Cd1—S1 in (**I**) is 2.540 (1) Å compared to 2.551 (1) Å in (**II**). The Cd—S bond lengths involving the bridging S atom (S2) in complex (**I**) are much longer that the terminal bonds, at 2.627 (1) and 2.841 (1) Å.

The difference in the Cd1—O bond lengths involving the di­methyl­formamide group in (**I**) and the dimethyl sulfoxide group in (**II**) is considerable; 2.275 (2) Å in (**I**) compared to 2.401 (2) Å in (**II**). However, a search of the CSD for compounds containing a Cd—O(DMF) or a Cd—O(DMSO) bond (with the following restrictions: three-dimensional coordinates determined, *R* factor ≤ 0.075, no disorder, no errors, not polymeric, no ions,and single crystals only) gave 37 hits for the former and 19 for the latter. The mean value for the Cd—O(DMF) bond length was found to be 2.33 (5) Å (varying from 2.225 to 2.482 Å). The mean value for the Cd—O(DMSO) bond length was found to be 2.33 (4) Å (varying from 2.25 to 2.412 Å). Thus, the Cd—O bond length observed for (**I**) is near the lower limit while the value observed for (**II**) is near the upper limit.

**Figure 2:**
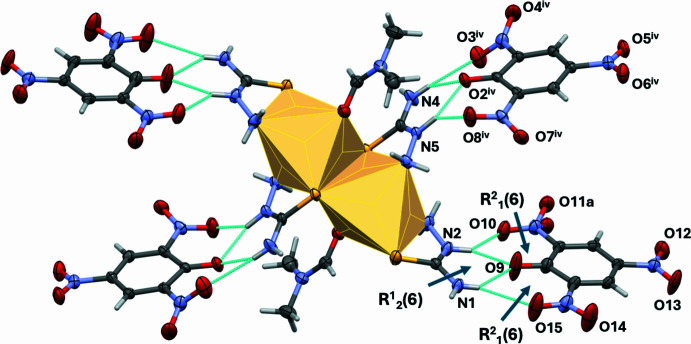
A view of the picrate anions hydrogen bonded to the complex cation (dashed lines, Table 2 ▸). The edge-sharing polyhedral of the cadmium(II) atoms are shown in yellow. The displacement ellipsoids are drawn at the 50% probability level. [Symmetry code: (iv) *x* + 1,*y*, *z*.]

In the picrate anions in (**I**) the nitro groups are inclined by different degrees to the phenolate rings to which they are attached: nitro groups N10/O3/O4, N11/O5/O6 and N12/O7/O8 are inclined to ring C6–C11 by 34.6 (5), 9.9 (4) and 19.3 (4)°, respectively, while nitro groups N13/O10/O11*A*, N14/O12/O13 and N15/O14/O15 are inclined to the C12–C17 ring by 18.0 (4), 4.8 (5) and 2.8 (6)°, respectively. The picrate anions accept N—H⋯O hydrogen bonds from the cation, as shown in Fig. 2 ▸ (see also Table 2 ▸). These hydrogen bonds, involving the phenolate O atoms (O2 and O9) and the adjacent nitro groups, are bifurcated, *viz*. two N—H⋯(O,O) links enclosing 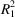
(6) ring motifs, which results in the central phenolate O atom acting as a double acceptor enclosing an 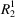
(6) motif. This situation was also observed in the crystal of complex (**II**), and in the crystal structure of thio­semicarbazidium picrate monohydrate mentioned above (YIFXUH; Xie, 2007 ▸).

## 3. Supra­molecular features

**Figure 3:**
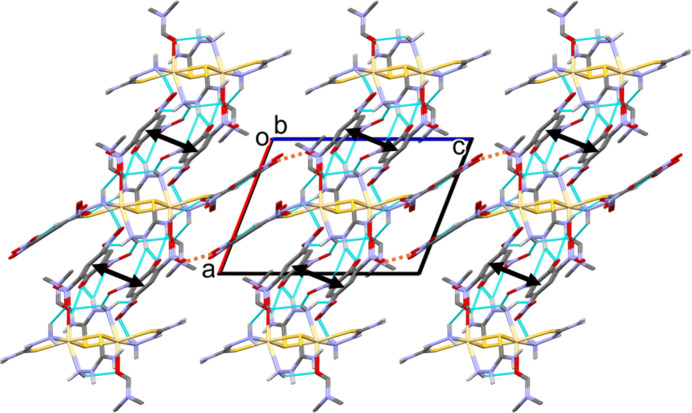
A view along the *b* axis of the crystal packing of compound (**I**). The N—H⋯O hydrogen bonds (Table 1 ▸) are shown as dashed lines. For clarity, H atoms not involved in hydrogen bonding have been omitted. The π–π inter­action is shown as a black double arrow and the C—H⋯O hydrogen bonds are shown as brown dashed lines.

In the crystal of (**I**), there are a large number of N—H⋯O hydrogen bonds present (Table 2 ▸). Apart from those noted above linking the complex cation and the picrate anions (Fig. 2 ▸) there are further N—H⋯O hydrogen bonds linking these units to form slabs lying parallel to the *ab* plane, as shown in Fig. 3 ▸. Within the slabs, parallel displaced π–π stacking inter­actions occur between inversion-related benzene rings (C6–C11) of a picrate anion: the centroid–centroid distance is 3.712 (2) Å, inter­planar distance = 3.375 (1) Å, slippage = 1.545 Å (shown in Fig. 3 ▸ as black double arrows). The slabs are linked by C—H⋯O hydrogen bonds (Table 2 ▸)

## 4. Hirshfeld surface analysis and two-dimensional fingerprint plots

**Figure 4:**
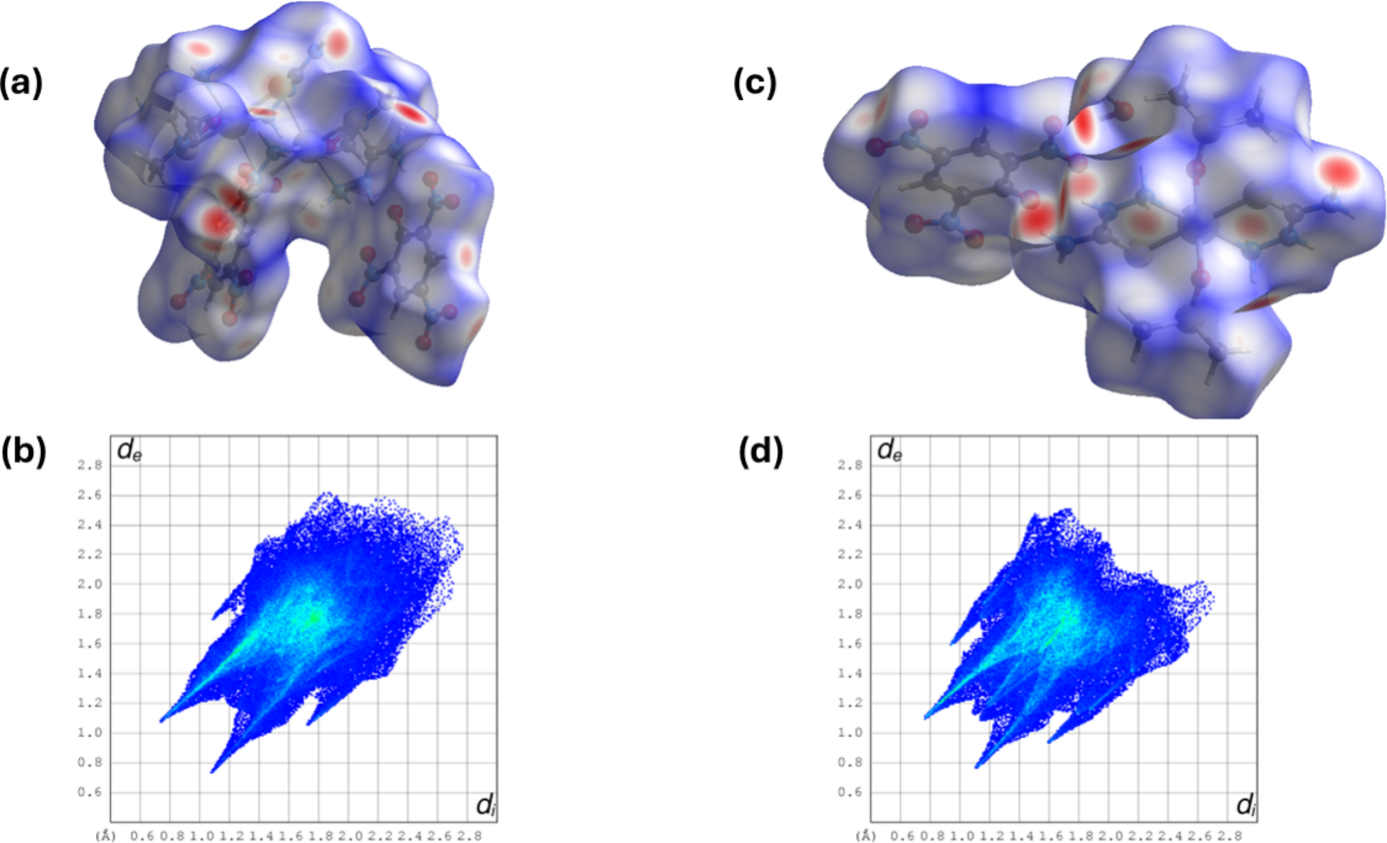
(*a*) The Hirshfeld surface of compound (**I**), mapped over *d*_norm_, (*b*) the full two-dimensional fingerprint plot for compound (**I**), (*c*) the Hirshfeld surface of compound (**II**), mapped over *d*_norm_ and (*d*) the full two-dimensional fingerprint plot for compound (**II**).

The Hirshfeld surface (HS) analyses and the associated two-dimensional fingerprint plots were performed with *CrystalExplorer17* (Spackman *et al.*, 2021 ▸) and inter­preted following the protocol of Tan *et al.* (2019 ▸). The Hirshfeld surfaces for compounds (**I**) and (**II**) are illustrated in Fig. 4 ▸*a* and 4*c*, respectively. A number of large red spots are observed in the HS which indicates that short contacts are highly significant in the crystal packing of both compounds.

**Figure 5:**
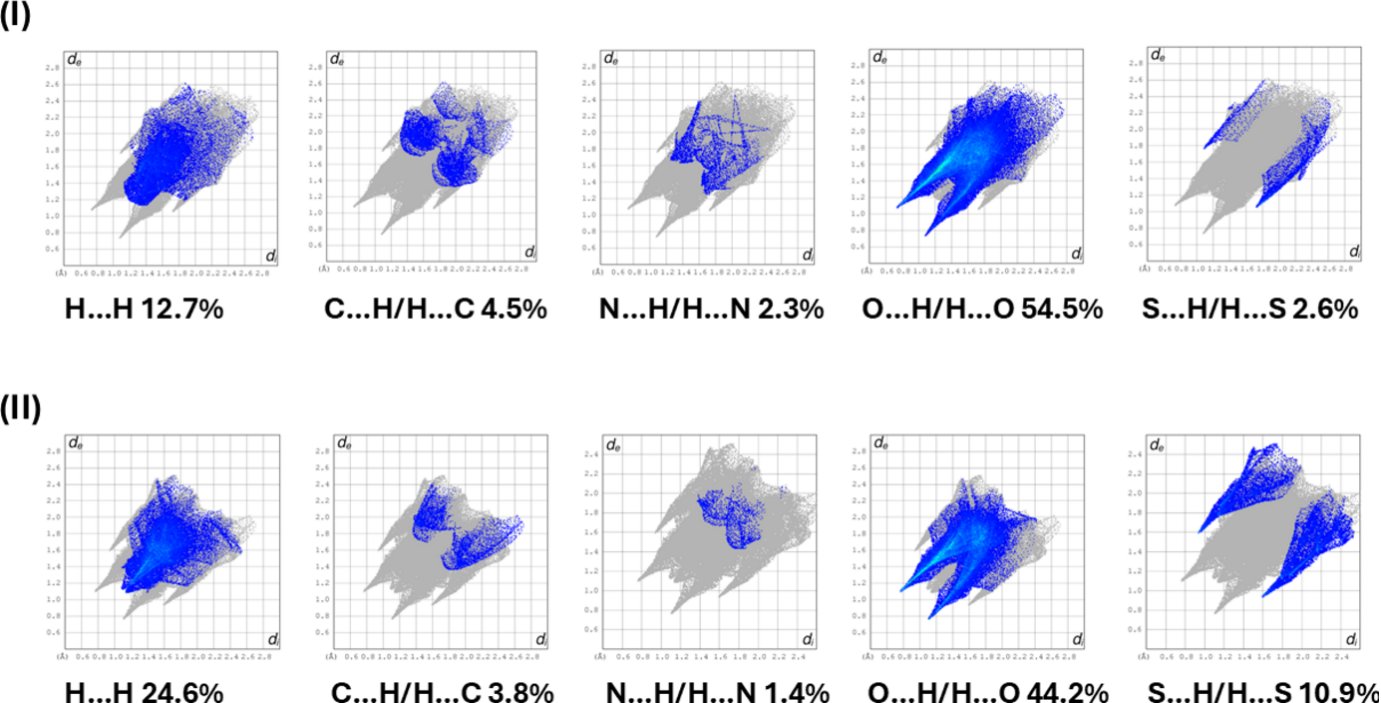
The principal two-dimensional fingerprint plots for compounds (**I**) and (**II**), delineated into H⋯H, C⋯H/H⋯C, N⋯H/H⋯O, O⋯H/H⋯O and S⋯H/H⋯S contacts.

The full two-dimensional fingerprint plots for compounds (**I**) and (**II**) are given in Fig. 4 ▸*b* and 4*d*, respectively. The principal percentage contributions of inter­atomic contacts to the Hirshfeld surfaces of (**I**) and (**II**) are compared in Table 3 ▸. Selected two-dimensional fingerprint plots for the two compounds are given in Fig. 5 ▸. For both crystal structures the major contributions are from O⋯H/H⋯O inter­actions; *viz*. 54.5% for (**I**) and 44.2% for (**II**). Both have sharp pincer-like spikes at *d*_e_*+ d*_i_ ≃ 1.85 Å. The H⋯H contacts also make significant contributions to the HS; 12.7% for (**I**) and 24.6% for (**II**). The S⋯H/H⋯S contributions are much more important for compound (**II**) at 10.9% than for compound (**I**) at 2.6%. Again both have sharp pincer-like spikes at *d*_e_*+ d*_i_ ≃ 2.8 Å for (**I)** and 2.5 Å for (**II**). These values can be correlated with the various hydrogen bonds and other inter­atomic inter­actions in the crystal (Table 2 ▸).

## 5. Synthesis and characterization

An equimolar ratio (1:1:1) of analytical grade reagents was used. Thio­semicarbazide (0.9 g) and CdCl_2_ (1.8 g) were dissolved in distilled water, then picric acid (2.3 g) dissolved in acetone was added under stirring. The mixture was refluxed at 373 K for 3 h, yielding a yellow crystalline precipitate. It was dissolved in DMF and a saturated solution at 303 K was prepared. The solvent was then allowed to evaporate slowly at room temperature, yielding large (*ca* 8 mm × 7 mm × 3 mm) yellow–orange block-like crystals of the title compound (**I**) [m.p. 412 (1) K], after a growth period of 32 days. Selected FTIR (KBr pellet, cm^−1^): 3419 (NH_2_ asymmetric stretch), 1643 (C=O stretch), 1265 (C—N stretch), 1082 (C=S stretch) (supporting information Fig. S1). For the UV/visible spectrum and TGA/DTA trace for (**I**) see Figs. S2 and S3 in the supporting information.

## 6. Refinement

Crystal data, data collection and structure refinement details are summarized in Table 4 ▸. The amine H atoms were located in difference-Fourier maps and were freely refined. Atom O11 of a picrate anion was modelled as disordered over two sites in a 0.85:0.15 ratio. The C-bound H atoms were included in calculated positions with C—H = 0.95–0.98 Å and *U*_iso_(H) = 1.2*U*_eq_(C) or 1.5*U*_eq_(methyl-C).

## Supplementary Material

Crystal structure: contains datablock(s) I, Global. DOI: 10.1107/S2056989025003974/hb8138sup1.cif

Structure factors: contains datablock(s) I. DOI: 10.1107/S2056989025003974/hb8138Isup2.hkl

Fig. S1 FTIR Spectrum for (I) FIG. S2 UV/visible spectrum for (I) FIG. S3 TGA/DTA trace for (I). DOI: 10.1107/S2056989025003974/hb8138sup3.pdf

CCDC reference: 2081043

Additional supporting information:  crystallographic information; 3D view; checkCIF report

## Figures and Tables

**Table 1:** A comparison of selected equivalent bond lengths (Å) and bond angles (°) in complexes (I) and (II)

Bond/angle	(**I**)	(**II**)^*a*^
Cd1—O1	2.275 (2)	2.401 (2)
Cd1—N3	2.398 (3)	2.382 (2)
Cd1—S1	2.540 (1)	2.551 (1)
		
Cd1—N6	2.421 (3)	–
Cd1—S2	2.627 (1)	–
Cd1—S1^i^	2.841 (1)	–
		
O1—Cd1—N6	160.89 (9)	180
S1—Cd1—S2	167.97 (3)	180
N3—Cd1—S2^ii^	171.43 (7)	180

Note: (*a*) Shanthakumari *et al.* (2011 ▸). Symmetry codes: (i) −*x* + 1, −*y* + 2, −*z* + 1; (ii) −*x* + 2, −*y*, −*z*.

**Table 2:** Hydrogen-bond geometry (Å, °)

*D*—H⋯*A*	*D*—H	H⋯*A*	*D*⋯*A*	*D*—H⋯*A*
N1—H1*AN*⋯O12^i^	0.86 (4)	2.06 (4)	2.908 (4)	168 (4)
N1—H1*BN*⋯O9	0.81 (5)	2.04 (5)	2.737 (4)	145 (4)
N1—H1*BN*⋯O15	0.81 (5)	2.46 (5)	3.130 (4)	141 (4)
N2—H2*N*⋯O9	0.94 (4)	1.94 (4)	2.721 (4)	139 (4)
N2—H2*N*⋯O10	0.94 (4)	2.19 (4)	2.992 (4)	142 (4)
N3—H3*AN*⋯O6^ii^	0.89 (6)	2.53 (6)	3.379 (5)	161 (5)
N3—H3*BN*⋯O3^iii^	0.96 (6)	2.32 (6)	3.018 (4)	130 (5)
N4—H4*AN*⋯O11*A*^ii^	0.78 (5)	2.36 (5)	3.060 (5)	149 (4)
N4—H4*BN*⋯O2^iv^	0.80 (5)	2.15 (5)	2.842 (4)	145 (5)
N4—H4*BN*⋯O3^iv^	0.80 (5)	2.43 (5)	3.079 (4)	139 (4)
N5—H5*N*⋯O2^iv^	0.95 (5)	1.88 (5)	2.753 (4)	151 (4)
N5—H5*N*⋯O8^iv^	0.95 (5)	2.42 (4)	3.156 (4)	134 (3)
N6—H6*AN*⋯O1^v^	0.85 (4)	2.48 (4)	3.116 (4)	132 (3)
N6—H6*AN*⋯O7^v^	0.85 (4)	2.27 (4)	2.966 (4)	139 (4)
N6—H6*BN*⋯O5^ii^	0.94 (5)	2.49 (5)	3.160 (4)	129 (4)
C5—H5*C*⋯O8^vi^	0.98	2.60	3.351 (5)	134
C16—H16⋯O4^vii^	0.95	2.46	3.374 (4)	161

Symmetry codes: (i) 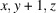; (ii) 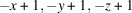; (iii) 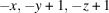; (iv) 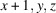; (v) 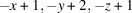; (vi) 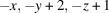; (vii) 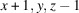.

**Table 3:** Principal percentage contributions of inter-atomic contacts to the Hirshfeld surfaces of compounds (I) and (II)^*a*^

Contact	(**I**)	(**II**)^*a*^
H⋯H	12.7	24.6
C⋯H/H⋯C	4.5	3.8
N⋯H/H⋯N	2.3	1.4
O⋯H/H⋯O	54.5	44.2
S⋯H/H⋯S	2.6	10.9
C⋯C	2.7	–
N⋯C/C⋯N	2.0	0.7
O⋯C/C⋯O	4.8	5.2
O⋯N/N⋯O	–	3.2
O⋯O	–	4.2
S⋯O/O⋯S	–	1.7

Note: (*a*) Shanthakumari *et al.* (2011 ▸).

**Table 4:** Experimental details

Crystal data	
Chemical formula	[Cd_2_(C_3_H_7_NO)_2_(CH_5_N_3_S)_4_](C_6_H_2_N_3_O_7_)_4_
*M*_r_	1647.97
Crystal system, space group	Triclinic, *P* 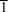
Temperature (K)	173
*a*, *b*, *c* (Å)	10.5367 (4), 10.9088 (5), 14.1593 (7)
α, β, γ (°)	92.782 (4), 109.796 (4), 104.077 (4)
*V* (Å^3^)	1469.98 (12)
*Z*	1
Radiation type	Cu *K*α
μ (mm^−1^)	8.14
Crystal size (mm)	0.50 × 0.35 × 0.25

Data collection	
Diffractometer	Xcalibur, Eos, Gemini
Absorption correction	Multi-scan (*CrysAlis PRO*; Rigaku OD, 2015 ▸)
*T*_min_, *T*_max_	0.326, 1.000
No. of measured, independent and observed [*I* > 2σ(*I*)] reflections	10301, 5601, 5028
*R*_int_	0.040
(sin θ/λ)_max_ (Å^−1^)	0.615

Refinement	
*R*[*F*^2^ > 2σ(*F*^2^)], *wR*(*F*^2^), *S*	0.037, 0.095, 1.02
No. of reflections	5601
No. of parameters	478
No. of restraints	1
H-atom treatment	H atoms treated by a mixture of independent and constrained refinement
Δρ_max_, Δρ_min_ (e Å^−3^)	0.91, −0.62

Computer programs: *CrysAlis PRO* (Rigaku OD, 2015 ▸), *SHELXT2019/3* (Sheldrick, 2015*a* ▸), *SHELXL2019/3* (Sheldrick, 2015*b* ▸), *PLATON* (Spek, 2020 ▸), *Mercury* (Macrae *et al.*, 2020 ▸) and *publCIF* (Westrip, 2010 ▸).

## supplementary crystallographic information

### Bis(µ-thiosemicarbazide-κ3N1,S:S;κ3S:N1,S)bis[(dimethylformamide-κO)(thiosemicarbazide-κ2N1,S)cadmium(II)] tetrakis(2,4,6-trinitrophenolate) Crystal data

**Table:**

[Cd_2_(C_3_H_7_NO)_2_(CH_5_N_3_S)_4_](C_6_H_2_N_3_O_7_)_4_	*Z* = 1
*M**_r_* = 1647.97	*F*(000) = 828
Triclinic, *P*1	*D*_x_ = 1.862 Mg m^−^^3^
*a* = 10.5367 (4) Å	Cu *K*α radiation, λ = 1.54184 Å
*b* = 10.9088 (5) Å	Cell parameters from 4951 reflections
*c* = 14.1593 (7) Å	θ = 4.2–71.4°
α = 92.782 (4)°	µ = 8.14 mm^−^^1^
β = 109.796 (4)°	*T* = 173 K
γ = 104.077 (4)°	Block, yellow
*V* = 1469.98 (12) Å^3^	0.50 × 0.35 × 0.25 mm

### Bis(µ-thiosemicarbazide-κ3N1,S:S;κ3S:N1,S)bis[(dimethylformamide-κO)(thiosemicarbazide-κ2N1,S)cadmium(II)] tetrakis(2,4,6-trinitrophenolate) Data collection

**Table:**

Xcalibur, Eos, Gemini diffractometer	5601 independent reflections
Radiation source: fine-focus sealed X-ray tube, Enhance (Cu) X-ray Source	5028 reflections with *I* > 2σ(*I*)
Graphite monochromator	*R*_int_ = 0.040
Detector resolution: 16.0416 pixels mm^-1^	θ_max_ = 71.4°, θ_min_ = 3.4°
ω scans	*h* = −8→12
Absorption correction: multi-scan (CrysAlisPro; Rigaku OD, 2015)	*k* = −12→13
*T*_min_ = 0.326, *T*_max_ = 1.000	*l* = −17→17
10301 measured reflections	

### Bis(µ-thiosemicarbazide-κ3N1,S:S;κ3S:N1,S)bis[(dimethylformamide-κO)(thiosemicarbazide-κ2N1,S)cadmium(II)] tetrakis(2,4,6-trinitrophenolate) Refinement

**Table:**

Refinement on *F*^2^	Primary atom site location: dual
Least-squares matrix: full	Secondary atom site location: difference Fourier map
*R*[*F*^2^ > 2σ(*F*^2^)] = 0.037	Hydrogen site location: mixed
*wR*(*F*^2^) = 0.095	H atoms treated by a mixture of independent and constrained refinement
*S* = 1.02	*w* = 1/[σ^2^(*F*_o_^2^) + (0.0524*P*)^2^] where *P* = (*F*_o_^2^ + 2*F*_c_^2^)/3
5601 reflections	(Δ/σ)_max_ = 0.001
478 parameters	Δρ_max_ = 0.91 e Å^−^^3^
1 restraint	Δρ_min_ = −0.62 e Å^−^^3^

### Bis(µ-thiosemicarbazide-κ3N1,S:S;κ3S:N1,S)bis[(dimethylformamide-κO)(thiosemicarbazide-κ2N1,S)cadmium(II)] tetrakis(2,4,6-trinitrophenolate) Special details

**Table:**

Geometry. All esds (except the esd in the dihedral angle between two l.s. planes) are estimated using the full covariance matrix. The cell esds are taken into account individually in the estimation of esds in distances, angles and torsion angles; correlations between esds in cell parameters are only used when they are defined by crystal symmetry. An approximate (isotropic) treatment of cell esds is used for estimating esds involving l.s. planes.

### Bis(µ-thiosemicarbazide-κ3N1,S:S;κ3S:N1,S)bis[(dimethylformamide-κO)(thiosemicarbazide-κ2N1,S)cadmium(II)] tetrakis(2,4,6-trinitrophenolate) Fractional atomic coordinates and isotropic or equivalent isotropic displacement parameters (Å^2^)

**Table:**

	*x*	*y*	*z*	*U*_iso_*/*U*_eq_	Occ. (<1)
Cd1	0.49782 (2)	0.92980 (2)	0.37745 (2)	0.02499 (9)	
S2	0.45606 (8)	0.81886 (7)	0.52909 (6)	0.02237 (16)	
S1	0.57145 (10)	1.00687 (8)	0.23246 (7)	0.03156 (19)	
O1	0.2648 (3)	0.9168 (2)	0.31788 (19)	0.0308 (5)	
O2	−0.0937 (3)	0.7022 (2)	0.6663 (2)	0.0303 (5)	
O3	−0.2412 (3)	0.4763 (3)	0.6984 (3)	0.0508 (8)	
O4	−0.0935 (4)	0.3885 (3)	0.7959 (3)	0.0495 (8)	
O5	0.2133 (3)	0.2920 (3)	0.6310 (2)	0.0401 (6)	
O6	0.3335 (3)	0.4463 (3)	0.5799 (3)	0.0427 (7)	
O7	0.1430 (3)	0.7964 (2)	0.4898 (2)	0.0334 (6)	
O8	0.0435 (4)	0.8716 (3)	0.5807 (3)	0.0474 (8)	
O9	0.6470 (5)	0.6190 (3)	0.1049 (4)	0.0795 (15)	
O10	0.4996 (3)	0.4696 (3)	0.1922 (3)	0.0463 (7)	
O11A	0.5513 (4)	0.2969 (3)	0.2271 (3)	0.0455 (9)	0.85
O11B	0.4768 (19)	0.262 (2)	0.1674 (14)	0.0455 (9)	0.15
O12	0.7272 (4)	0.0845 (3)	0.0154 (3)	0.0470 (7)	
O13	0.8366 (5)	0.1917 (3)	−0.0681 (3)	0.0688 (12)	
O14	0.8799 (5)	0.6291 (3)	−0.0750 (4)	0.0791 (15)	
O15	0.7789 (5)	0.7313 (3)	−0.0066 (4)	0.0820 (16)	
N1	0.6282 (3)	0.8649 (3)	0.1059 (2)	0.0290 (6)	
H1AN	0.658 (4)	0.937 (4)	0.087 (3)	0.026 (10)*	
H1BN	0.639 (5)	0.801 (4)	0.083 (4)	0.034 (12)*	
N2	0.5270 (3)	0.7499 (3)	0.2023 (2)	0.0271 (6)	
H2N	0.539 (4)	0.673 (4)	0.178 (3)	0.032 (11)*	
N3	0.4735 (4)	0.7328 (3)	0.2812 (2)	0.0315 (6)	
H3AN	0.520 (6)	0.695 (5)	0.330 (4)	0.053 (15)*	
H3BN	0.375 (6)	0.693 (5)	0.253 (5)	0.067 (17)*	
N4	0.6257 (4)	0.6970 (3)	0.6412 (3)	0.0371 (8)	
H4AN	0.567 (5)	0.671 (4)	0.663 (3)	0.028 (11)*	
H4BN	0.694 (5)	0.673 (4)	0.661 (4)	0.041 (13)*	
N5	0.7261 (3)	0.8318 (3)	0.5551 (2)	0.0222 (5)	
H5N	0.809 (5)	0.805 (4)	0.585 (4)	0.037 (12)*	
N6	0.7277 (3)	0.9252 (3)	0.4900 (2)	0.0266 (6)	
H6AN	0.762 (4)	0.998 (4)	0.526 (3)	0.023 (10)*	
H6BN	0.787 (6)	0.912 (5)	0.456 (4)	0.057 (15)*	
N7	0.0868 (3)	0.9859 (3)	0.2160 (2)	0.0331 (7)	
N10	−0.1256 (4)	0.4557 (3)	0.7304 (3)	0.0328 (7)	
N11	0.2397 (3)	0.4025 (3)	0.6116 (2)	0.0288 (6)	
N12	0.0900 (3)	0.7869 (3)	0.5553 (2)	0.0261 (6)	
N13	0.5526 (3)	0.3869 (3)	0.1780 (2)	0.0335 (7)	
N14	0.7719 (4)	0.1851 (3)	−0.0113 (2)	0.0335 (7)	
N15	0.8085 (4)	0.6347 (3)	−0.0242 (3)	0.0396 (8)	
C2	0.6139 (3)	0.7811 (3)	0.5782 (2)	0.0230 (6)	
C1	0.5757 (3)	0.8647 (3)	0.1789 (2)	0.0231 (6)	
C3	0.2170 (4)	0.9789 (3)	0.2489 (3)	0.0299 (7)	
H3	0.278573	1.023812	0.218513	0.036*	
C4	0.0378 (6)	1.0605 (5)	0.1359 (3)	0.0553 (13)	
H4C	−0.002629	1.122138	0.159607	0.083*	
H4B	−0.033805	1.003453	0.076192	0.083*	
H4A	0.116864	1.106238	0.118002	0.083*	
C5	−0.0162 (4)	0.9147 (4)	0.2543 (3)	0.0438 (10)	
H5C	−0.072361	0.969362	0.265949	0.066*	
H5B	0.031938	0.886843	0.318241	0.066*	
H5A	−0.077833	0.839869	0.204584	0.066*	
C6	−0.0197 (3)	0.6351 (3)	0.6521 (2)	0.0213 (6)	
C7	−0.0230 (3)	0.5117 (3)	0.6852 (2)	0.0231 (6)	
C8	0.0616 (4)	0.4384 (3)	0.6752 (2)	0.0237 (6)	
H8	0.056381	0.359107	0.700753	0.028*	
C9	0.1550 (3)	0.4822 (3)	0.6270 (2)	0.0237 (6)	
C10	0.1650 (3)	0.5977 (3)	0.5895 (2)	0.0229 (6)	
H10	0.229084	0.625979	0.556155	0.027*	
C11	0.0801 (3)	0.6710 (3)	0.6016 (2)	0.0221 (6)	
C12	0.6766 (4)	0.5218 (3)	0.0800 (3)	0.0334 (8)	
C13	0.6341 (4)	0.3998 (3)	0.1128 (2)	0.0265 (7)	
C14	0.6667 (4)	0.2920 (3)	0.0858 (2)	0.0266 (7)	
H14	0.636851	0.214241	0.109585	0.032*	
C15	0.7442 (4)	0.2991 (3)	0.0231 (2)	0.0267 (7)	
C16	0.7908 (4)	0.4120 (3)	−0.0109 (3)	0.0296 (7)	
H16	0.844751	0.415206	−0.052997	0.035*	
C17	0.7584 (4)	0.5193 (3)	0.0167 (3)	0.0291 (7)	

### Bis(µ-thiosemicarbazide-κ3N1,S:S;κ3S:N1,S)bis[(dimethylformamide-κO)(thiosemicarbazide-κ2N1,S)cadmium(II)] tetrakis(2,4,6-trinitrophenolate) Atomic displacement parameters (Å^2^)

**Table:**

	*U*^11^	*U*^22^	*U*^33^	*U*^12^	*U*^13^	*U*^23^
Cd1	0.02764 (13)	0.03138 (14)	0.02463 (12)	0.01650 (10)	0.01366 (9)	0.00976 (9)
S2	0.0217 (4)	0.0235 (4)	0.0299 (4)	0.0115 (3)	0.0147 (3)	0.0105 (3)
S1	0.0489 (5)	0.0241 (4)	0.0362 (4)	0.0169 (4)	0.0277 (4)	0.0120 (3)
O1	0.0303 (12)	0.0381 (14)	0.0305 (12)	0.0176 (11)	0.0127 (10)	0.0105 (10)
O2	0.0305 (12)	0.0322 (13)	0.0422 (14)	0.0192 (10)	0.0216 (11)	0.0180 (11)
O3	0.0398 (16)	0.0476 (18)	0.085 (2)	0.0166 (13)	0.0415 (17)	0.0299 (16)
O4	0.081 (2)	0.0456 (17)	0.0540 (18)	0.0340 (16)	0.0493 (18)	0.0331 (15)
O5	0.0513 (17)	0.0289 (14)	0.0545 (17)	0.0249 (12)	0.0266 (14)	0.0136 (12)
O6	0.0418 (16)	0.0397 (16)	0.0624 (19)	0.0181 (13)	0.0335 (15)	0.0086 (13)
O7	0.0349 (13)	0.0360 (14)	0.0421 (14)	0.0135 (11)	0.0252 (12)	0.0227 (11)
O8	0.071 (2)	0.0298 (14)	0.071 (2)	0.0283 (14)	0.0490 (18)	0.0252 (14)
O9	0.144 (4)	0.0351 (17)	0.130 (4)	0.042 (2)	0.121 (4)	0.033 (2)
O10	0.0597 (19)	0.0401 (16)	0.0604 (19)	0.0188 (14)	0.0445 (16)	0.0091 (14)
O11A	0.072 (3)	0.0412 (19)	0.047 (2)	0.0263 (18)	0.0412 (19)	0.0214 (16)
O11B	0.072 (3)	0.0412 (19)	0.047 (2)	0.0263 (18)	0.0412 (19)	0.0214 (16)
O12	0.073 (2)	0.0297 (15)	0.0609 (19)	0.0245 (14)	0.0424 (17)	0.0223 (13)
O13	0.131 (4)	0.0371 (17)	0.087 (3)	0.032 (2)	0.093 (3)	0.0160 (17)
O14	0.135 (4)	0.0392 (18)	0.121 (4)	0.029 (2)	0.112 (3)	0.030 (2)
O15	0.152 (4)	0.0304 (17)	0.125 (4)	0.036 (2)	0.117 (4)	0.029 (2)
N1	0.0399 (17)	0.0256 (16)	0.0333 (15)	0.0129 (13)	0.0244 (13)	0.0086 (13)
N2	0.0352 (15)	0.0225 (14)	0.0309 (14)	0.0085 (12)	0.0205 (12)	0.0057 (11)
N3	0.0389 (17)	0.0323 (16)	0.0347 (16)	0.0104 (14)	0.0262 (14)	0.0106 (13)
N4	0.0345 (17)	0.048 (2)	0.055 (2)	0.0279 (16)	0.0330 (16)	0.0379 (17)
N5	0.0242 (13)	0.0223 (14)	0.0288 (13)	0.0121 (11)	0.0152 (11)	0.0109 (11)
N6	0.0279 (14)	0.0244 (15)	0.0345 (15)	0.0108 (12)	0.0164 (12)	0.0135 (13)
N7	0.0381 (16)	0.0392 (17)	0.0243 (14)	0.0219 (14)	0.0063 (12)	0.0053 (12)
N10	0.0448 (18)	0.0251 (15)	0.0411 (17)	0.0130 (13)	0.0280 (15)	0.0112 (13)
N11	0.0319 (15)	0.0295 (16)	0.0304 (14)	0.0159 (12)	0.0132 (12)	0.0041 (12)
N12	0.0247 (13)	0.0223 (14)	0.0340 (14)	0.0074 (11)	0.0122 (12)	0.0106 (11)
N13	0.0428 (18)	0.0334 (17)	0.0384 (16)	0.0170 (14)	0.0265 (14)	0.0142 (13)
N14	0.0502 (19)	0.0289 (16)	0.0320 (15)	0.0174 (14)	0.0226 (14)	0.0097 (12)
N15	0.059 (2)	0.0261 (16)	0.0455 (18)	0.0097 (15)	0.0352 (17)	0.0063 (14)
C2	0.0263 (16)	0.0221 (16)	0.0288 (15)	0.0137 (13)	0.0147 (13)	0.0077 (12)
C1	0.0221 (15)	0.0248 (16)	0.0250 (15)	0.0096 (12)	0.0093 (12)	0.0073 (12)
C3	0.0370 (19)	0.0331 (19)	0.0243 (15)	0.0142 (15)	0.0136 (14)	0.0044 (13)
C4	0.073 (3)	0.062 (3)	0.034 (2)	0.040 (3)	0.007 (2)	0.015 (2)
C5	0.0297 (19)	0.057 (3)	0.045 (2)	0.0168 (18)	0.0101 (17)	0.0080 (19)
C6	0.0199 (14)	0.0252 (16)	0.0202 (13)	0.0108 (12)	0.0053 (11)	0.0079 (12)
C7	0.0261 (16)	0.0249 (16)	0.0214 (14)	0.0090 (13)	0.0106 (12)	0.0079 (12)
C8	0.0312 (16)	0.0209 (16)	0.0220 (14)	0.0105 (13)	0.0104 (13)	0.0061 (12)
C9	0.0246 (15)	0.0248 (16)	0.0252 (15)	0.0125 (13)	0.0090 (13)	0.0068 (12)
C10	0.0219 (14)	0.0250 (16)	0.0233 (14)	0.0052 (12)	0.0109 (12)	0.0047 (12)
C11	0.0207 (14)	0.0199 (15)	0.0259 (15)	0.0072 (12)	0.0069 (12)	0.0080 (12)
C12	0.046 (2)	0.0261 (18)	0.0379 (19)	0.0117 (15)	0.0259 (17)	0.0063 (14)
C13	0.0328 (17)	0.0308 (18)	0.0222 (15)	0.0130 (14)	0.0143 (13)	0.0066 (13)
C14	0.0320 (17)	0.0287 (17)	0.0213 (14)	0.0095 (14)	0.0109 (13)	0.0081 (12)
C15	0.0370 (18)	0.0276 (17)	0.0214 (14)	0.0138 (14)	0.0141 (13)	0.0064 (12)
C16	0.0396 (19)	0.0324 (19)	0.0231 (15)	0.0118 (15)	0.0180 (14)	0.0047 (13)
C17	0.0424 (19)	0.0217 (16)	0.0277 (16)	0.0060 (14)	0.0201 (15)	0.0030 (13)

### Bis(µ-thiosemicarbazide-κ3N1,S:S;κ3S:N1,S)bis[(dimethylformamide-κO)(thiosemicarbazide-κ2N1,S)cadmium(II)] tetrakis(2,4,6-trinitrophenolate) Geometric parameters (Å, º)

**Table:**

Cd1—O1	2.275 (2)	N5—N6	1.407 (4)
Cd1—N3	2.398 (3)	N5—H5N	0.95 (5)
Cd1—N6	2.421 (3)	N6—H6AN	0.85 (4)
Cd1—S1	2.5402 (9)	N6—H6BN	0.94 (5)
Cd1—S2	2.6271 (8)	N7—C3	1.314 (5)
Cd1—S2^i^	2.8406 (8)	N7—C5	1.451 (5)
S2—C2	1.731 (3)	N7—C4	1.453 (5)
S1—C1	1.711 (3)	N10—C7	1.462 (4)
O1—C3	1.250 (4)	N11—C9	1.446 (4)
O2—C6	1.247 (4)	N12—C11	1.449 (4)
O3—N10	1.228 (5)	N13—C13	1.449 (4)
O4—N10	1.219 (4)	N14—C15	1.444 (4)
O5—N11	1.236 (4)	N15—C17	1.467 (5)
O6—N11	1.223 (4)	C3—H3	0.9500
O7—N12	1.230 (4)	C4—H4C	0.9800
O8—N12	1.237 (4)	C4—H4B	0.9800
O9—C12	1.242 (5)	C4—H4A	0.9800
O10—N13	1.213 (4)	C5—H5C	0.9800
O11A—N13	1.230 (5)	C5—H5B	0.9800
O11B—N13	1.37 (2)	C5—H5A	0.9800
O12—N14	1.216 (4)	C6—C7	1.442 (4)
O13—N14	1.212 (4)	C6—C11	1.452 (5)
O14—N15	1.212 (5)	C7—C8	1.370 (5)
O15—N15	1.207 (5)	C8—C9	1.386 (5)
N1—C1	1.326 (4)	C8—H8	0.9500
N1—H1AN	0.86 (4)	C9—C10	1.385 (5)
N1—H1BN	0.81 (5)	C10—C11	1.382 (4)
N2—C1	1.336 (4)	C10—H10	0.9500
N2—N3	1.414 (4)	C12—C17	1.444 (5)
N2—H2N	0.94 (4)	C12—C13	1.448 (5)
N3—H3AN	0.89 (6)	C13—C14	1.374 (5)
N3—H3BN	0.96 (6)	C14—C15	1.389 (5)
N4—C2	1.310 (4)	C14—H14	0.9500
N4—H4AN	0.78 (5)	C15—C16	1.383 (5)
N4—H4BN	0.80 (5)	C16—C17	1.370 (5)
N5—C2	1.331 (4)	C16—H16	0.9500
			
O1—Cd1—N3	95.40 (11)	O13—N14—C15	119.0 (3)
O1—Cd1—N6	160.89 (9)	O12—N14—C15	119.3 (3)
N3—Cd1—N6	90.03 (12)	O15—N15—O14	122.1 (3)
O1—Cd1—S1	102.28 (7)	O15—N15—C17	120.0 (3)
N3—Cd1—S1	77.96 (7)	O14—N15—C17	117.9 (3)
N6—Cd1—S1	96.77 (7)	N4—C2—N5	116.9 (3)
O1—Cd1—S2	86.86 (6)	N4—C2—S2	119.1 (3)
N3—Cd1—S2	93.55 (7)	N5—C2—S2	124.0 (2)
N6—Cd1—S2	74.50 (7)	N1—C1—N2	115.6 (3)
S1—Cd1—S2	167.97 (3)	N1—C1—S1	118.8 (3)
O1—Cd1—S2^i^	87.19 (7)	N2—C1—S1	125.6 (3)
N3—Cd1—S2^i^	171.43 (7)	O1—C3—N7	124.1 (3)
N6—Cd1—S2^i^	90.14 (8)	O1—C3—H3	118.0
S1—Cd1—S2^i^	93.51 (3)	N7—C3—H3	118.0
S2—Cd1—S2^i^	94.75 (2)	N7—C4—H4C	109.5
C2—S2—Cd1	98.61 (11)	N7—C4—H4B	109.5
C2—S2—Cd1^i^	106.39 (11)	H4C—C4—H4B	109.5
Cd1—S2—Cd1^i^	85.25 (2)	N7—C4—H4A	109.5
C1—S1—Cd1	98.99 (11)	H4C—C4—H4A	109.5
C3—O1—Cd1	119.9 (2)	H4B—C4—H4A	109.5
C1—N1—H1AN	118 (3)	N7—C5—H5C	109.5
C1—N1—H1BN	121 (3)	N7—C5—H5B	109.5
H1AN—N1—H1BN	120 (4)	H5C—C5—H5B	109.5
C1—N2—N3	123.0 (3)	N7—C5—H5A	109.5
C1—N2—H2N	125 (3)	H5C—C5—H5A	109.5
N3—N2—H2N	111 (3)	H5B—C5—H5A	109.5
N2—N3—Cd1	113.3 (2)	O2—C6—C7	123.4 (3)
N2—N3—H3AN	114 (3)	O2—C6—C11	124.6 (3)
Cd1—N3—H3AN	101 (3)	C7—C6—C11	111.9 (3)
N2—N3—H3BN	110 (4)	C8—C7—C6	124.9 (3)
Cd1—N3—H3BN	105 (3)	C8—C7—N10	115.6 (3)
H3AN—N3—H3BN	113 (5)	C6—C7—N10	119.4 (3)
C2—N4—H4AN	121 (3)	C7—C8—C9	118.6 (3)
C2—N4—H4BN	122 (4)	C7—C8—H8	120.7
H4AN—N4—H4BN	117 (5)	C9—C8—H8	120.7
C2—N5—N6	122.3 (3)	C10—C9—C8	121.7 (3)
C2—N5—H5N	119 (3)	C10—C9—N11	119.2 (3)
N6—N5—H5N	119 (3)	C8—C9—N11	119.0 (3)
N5—N6—Cd1	114.5 (2)	C11—C10—C9	118.8 (3)
N5—N6—H6AN	108 (3)	C11—C10—H10	120.6
Cd1—N6—H6AN	104 (3)	C9—C10—H10	120.6
N5—N6—H6BN	106 (3)	C10—C11—N12	115.8 (3)
Cd1—N6—H6BN	114 (3)	C10—C11—C6	124.0 (3)
H6AN—N6—H6BN	110 (4)	N12—C11—C6	120.2 (3)
C3—N7—C5	121.6 (3)	O9—C12—C17	123.5 (3)
C3—N7—C4	121.4 (4)	O9—C12—C13	123.5 (3)
C5—N7—C4	117.0 (4)	C17—C12—C13	113.0 (3)
O4—N10—O3	123.5 (3)	C14—C13—C12	123.8 (3)
O4—N10—C7	118.5 (3)	C14—C13—N13	116.4 (3)
O3—N10—C7	118.0 (3)	C12—C13—N13	119.9 (3)
O6—N11—O5	122.8 (3)	C13—C14—C15	118.7 (3)
O6—N11—C9	119.2 (3)	C13—C14—H14	120.7
O5—N11—C9	117.9 (3)	C15—C14—H14	120.7
O7—N12—O8	121.9 (3)	C16—C15—C14	121.5 (3)
O7—N12—C11	118.1 (3)	C16—C15—N14	118.9 (3)
O8—N12—C11	119.9 (3)	C14—C15—N14	119.5 (3)
O10—N13—O11A	120.5 (3)	C17—C16—C15	119.5 (3)
O10—N13—O11B	117.9 (8)	C17—C16—H16	120.3
O10—N13—C13	120.9 (3)	C15—C16—H16	120.3
O11A—N13—C13	118.2 (3)	C16—C17—C12	123.5 (3)
O11B—N13—C13	110.4 (9)	C16—C17—N15	116.4 (3)
O13—N14—O12	121.7 (3)	C12—C17—N15	120.1 (3)
			
C1—N2—N3—Cd1	−5.1 (4)	O7—N12—C11—C6	−159.4 (3)
C2—N5—N6—Cd1	−21.7 (4)	O8—N12—C11—C6	19.2 (5)
N6—N5—C2—N4	−178.6 (3)	O2—C6—C11—C10	177.6 (3)
N6—N5—C2—S2	2.1 (5)	C7—C6—C11—C10	−2.0 (5)
Cd1—S2—C2—N4	−163.7 (3)	O2—C6—C11—N12	−5.7 (5)
Cd1^i^—S2—C2—N4	108.7 (3)	C7—C6—C11—N12	174.8 (3)
Cd1—S2—C2—N5	15.6 (3)	O9—C12—C13—C14	−179.8 (5)
Cd1^i^—S2—C2—N5	−72.0 (3)	C17—C12—C13—C14	−0.6 (5)
N3—N2—C1—N1	177.0 (3)	O9—C12—C13—N13	0.0 (7)
N3—N2—C1—S1	−4.6 (5)	C17—C12—C13—N13	179.2 (3)
Cd1—S1—C1—N1	−171.4 (3)	O10—N13—C13—C14	−167.9 (4)
Cd1—S1—C1—N2	10.4 (3)	O11A—N13—C13—C14	19.9 (5)
Cd1—O1—C3—N7	−173.9 (3)	O11B—N13—C13—C14	−24.5 (8)
C5—N7—C3—O1	−3.4 (6)	O10—N13—C13—C12	12.3 (6)
C4—N7—C3—O1	179.6 (4)	O11A—N13—C13—C12	−159.9 (4)
O2—C6—C7—C8	−176.7 (3)	O11B—N13—C13—C12	155.7 (8)
C11—C6—C7—C8	2.9 (5)	C12—C13—C14—C15	−0.4 (5)
O2—C6—C7—N10	5.3 (5)	N13—C13—C14—C15	179.8 (3)
C11—C6—C7—N10	−175.1 (3)	C13—C14—C15—C16	1.2 (5)
O4—N10—C7—C8	34.2 (5)	C13—C14—C15—N14	−176.1 (3)
O3—N10—C7—C8	−144.0 (3)	O13—N14—C15—C16	0.8 (6)
O4—N10—C7—C6	−147.5 (3)	O12—N14—C15—C16	−177.5 (4)
O3—N10—C7—C6	34.2 (5)	O13—N14—C15—C14	178.2 (4)
C6—C7—C8—C9	−2.2 (5)	O12—N14—C15—C14	−0.2 (5)
N10—C7—C8—C9	175.9 (3)	C14—C15—C16—C17	−0.9 (5)
C7—C8—C9—C10	0.3 (5)	N14—C15—C16—C17	176.4 (3)
C7—C8—C9—N11	−176.9 (3)	C15—C16—C17—C12	−0.2 (6)
O6—N11—C9—C10	11.0 (5)	C15—C16—C17—N15	−178.6 (3)
O5—N11—C9—C10	−168.7 (3)	O9—C12—C17—C16	−179.9 (5)
O6—N11—C9—C8	−171.6 (3)	C13—C12—C17—C16	0.9 (6)
O5—N11—C9—C8	8.6 (5)	O9—C12—C17—N15	−1.6 (7)
C8—C9—C10—C11	0.5 (5)	C13—C12—C17—N15	179.2 (3)
N11—C9—C10—C11	177.8 (3)	O15—N15—C17—C16	176.9 (5)
C9—C10—C11—N12	−176.5 (3)	O14—N15—C17—C16	−3.4 (6)
C9—C10—C11—C6	0.4 (5)	O15—N15—C17—C12	−1.5 (6)
O7—N12—C11—C10	17.6 (4)	O14—N15—C17—C12	178.2 (5)
O8—N12—C11—C10	−163.8 (3)		

Symmetry code: (i) −*x*+1, −*y*+2, −*z*+1.

### Bis(µ-thiosemicarbazide-κ3N1,S:S;κ3S:N1,S)bis[(dimethylformamide-κO)(thiosemicarbazide-κ2N1,S)cadmium(II)] tetrakis(2,4,6-trinitrophenolate) Hydrogen-bond geometry (Å, º)

**Table:**

*D*—H···*A*	*D*—H	H···*A*	*D*···*A*	*D*—H···*A*
N1—H1*AN*···O12^ii^	0.86 (4)	2.06 (4)	2.908 (4)	168 (4)
N1—H1*BN*···O9	0.81 (5)	2.04 (5)	2.737 (4)	145 (4)
N1—H1*BN*···O15	0.81 (5)	2.46 (5)	3.130 (4)	141 (4)
N2—H2*N*···O9	0.94 (4)	1.94 (4)	2.721 (4)	139 (4)
N2—H2*N*···O10	0.94 (4)	2.19 (4)	2.992 (4)	142 (4)
N3—H3*AN*···O6^iii^	0.89 (6)	2.53 (6)	3.379 (5)	161 (5)
N3—H3*BN*···O3^iv^	0.96 (6)	2.32 (6)	3.018 (4)	130 (5)
N4—H4*AN*···O11*A*^iii^	0.78 (5)	2.36 (5)	3.060 (5)	149 (4)
N4—H4*BN*···O2^v^	0.80 (5)	2.15 (5)	2.842 (4)	145 (5)
N4—H4*BN*···O3^v^	0.80 (5)	2.43 (5)	3.079 (4)	139 (4)
N5—H5*N*···O2^v^	0.95 (5)	1.88 (5)	2.753 (4)	151 (4)
N5—H5*N*···O8^v^	0.95 (5)	2.42 (4)	3.156 (4)	134 (3)
N6—H6*AN*···O1^i^	0.85 (4)	2.48 (4)	3.116 (4)	132 (3)
N6—H6*AN*···O7^i^	0.85 (4)	2.27 (4)	2.966 (4)	139 (4)
N6—H6*BN*···O5^iii^	0.94 (5)	2.49 (5)	3.160 (4)	129 (4)
C5—H5*C*···O8^vi^	0.98	2.60	3.351 (5)	134
C16—H16···O4^vii^	0.95	2.46	3.374 (4)	161

Symmetry codes: (i) −*x*+1, −*y*+2, −*z*+1; (ii) *x*, *y*+1, *z*; (iii) −*x*+1, −*y*+1, −*z*+1; (iv) −*x*, −*y*+1, −*z*+1; (v) *x*+1, *y*, *z*; (vi) −*x*, −*y*+2, −*z*+1; (vii) *x*+1, *y*, *z*−1.
