# Supplementary material for: Bis(μ-thio­semicarbazide-κ3N1,S:S;κ3S:N1,S)bis­[(di­methyl­formamide-κO)(thio­semicarbazide-κ2N1,S)cadmium(II)] tetra­kis­(2,4,6-tri­nitro­phen­olate): synthesis, crystal structure and Hirshfeld surface analysis
Source: Acta Crystallogr E Crystallogr Commun. 2025 May 13;81(Pt 6):505–9. doi: 10.1107/S2056989025003974 (PMC12142421; doi:10.1107/S2056989025003974)
Supplement: Supplementary file 3 [file e-81-00505-sup3.pdf]

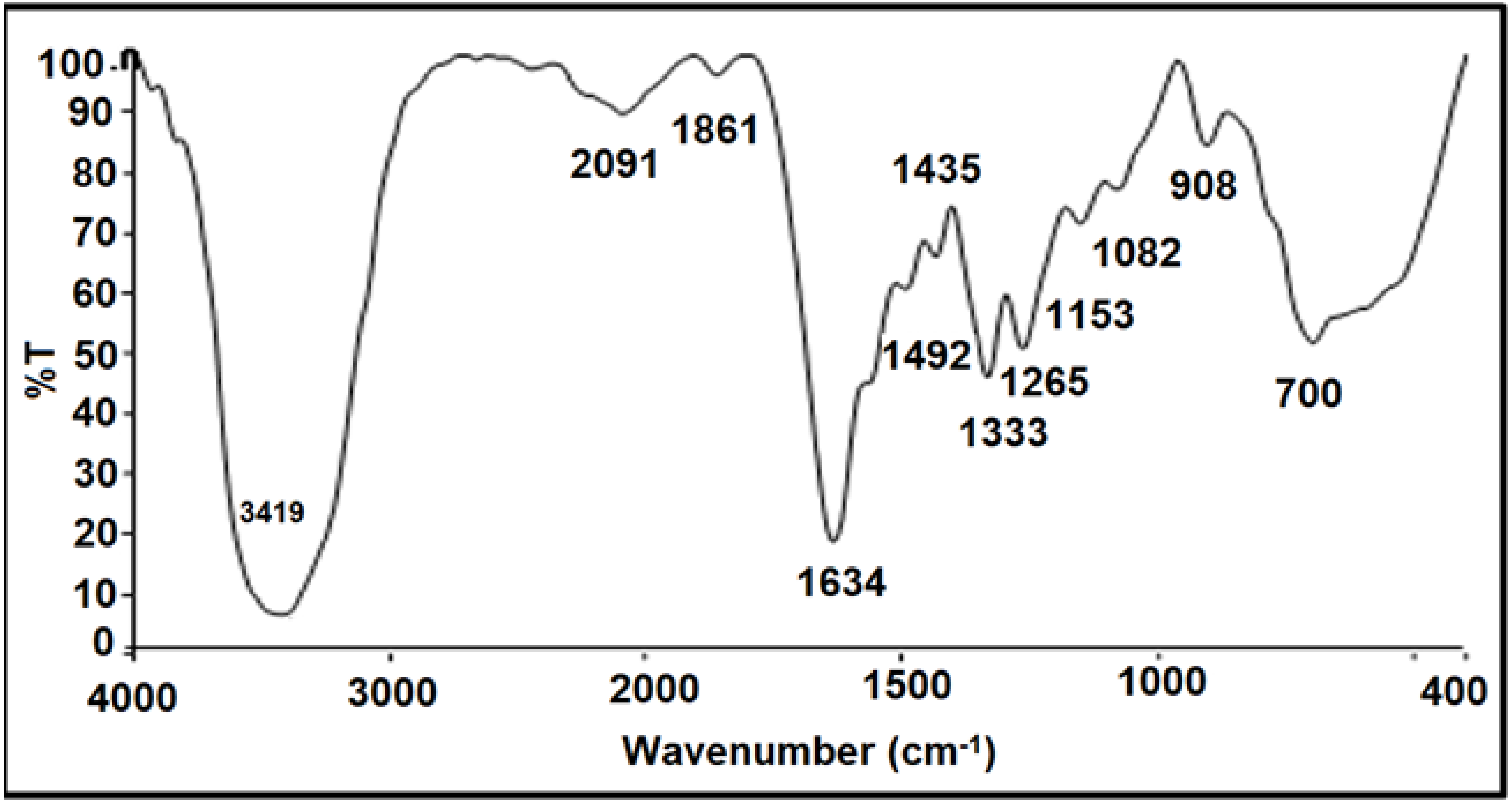

Fig. S1 Fourier Transform Infrared (FTIR) spectrum of (I) recorded in the range 400-4000 cm^{-1}

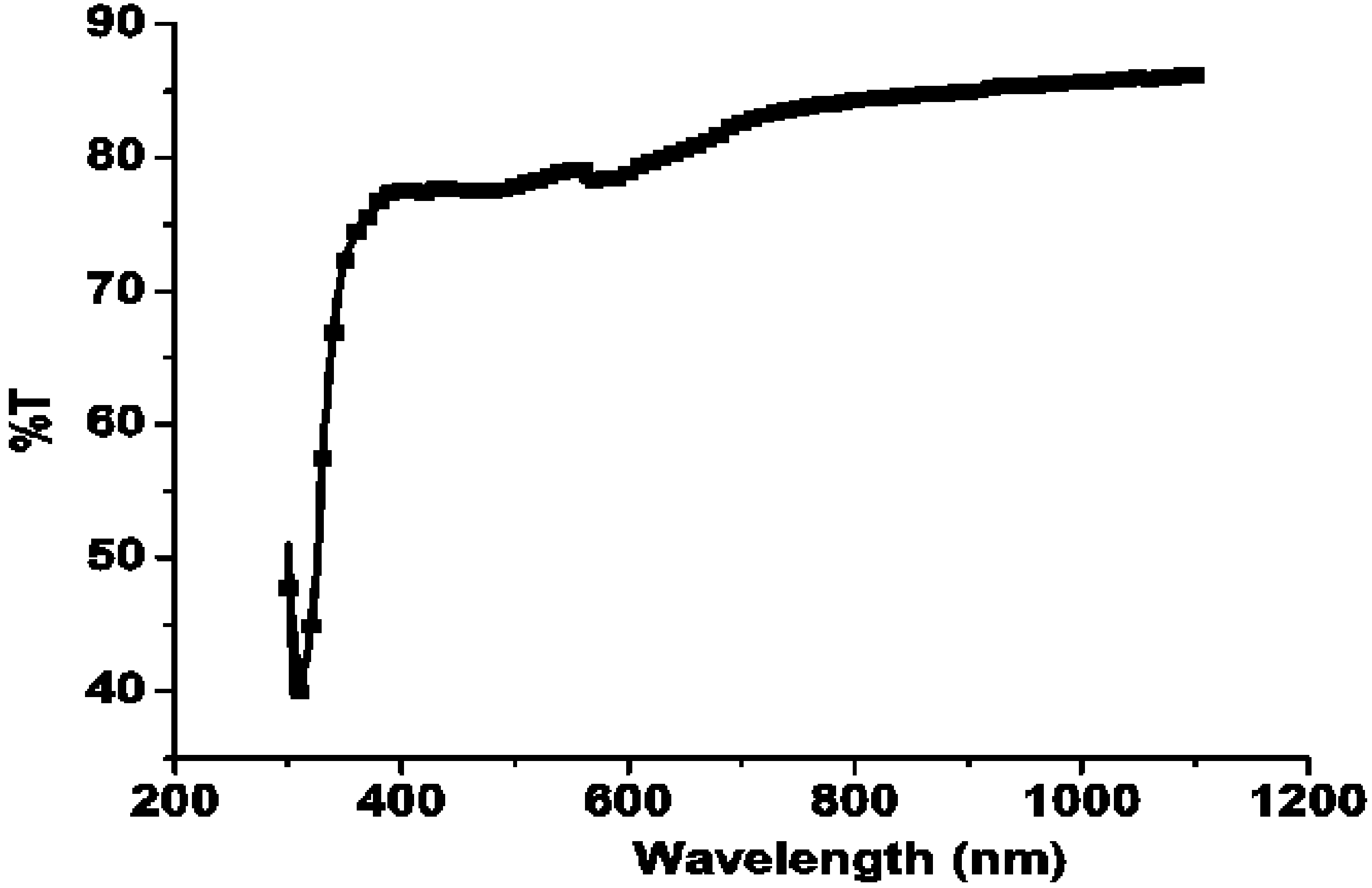

Fig. S2 The optical transmission spectrum of (I) recorded in the range 200 – 1100 nm

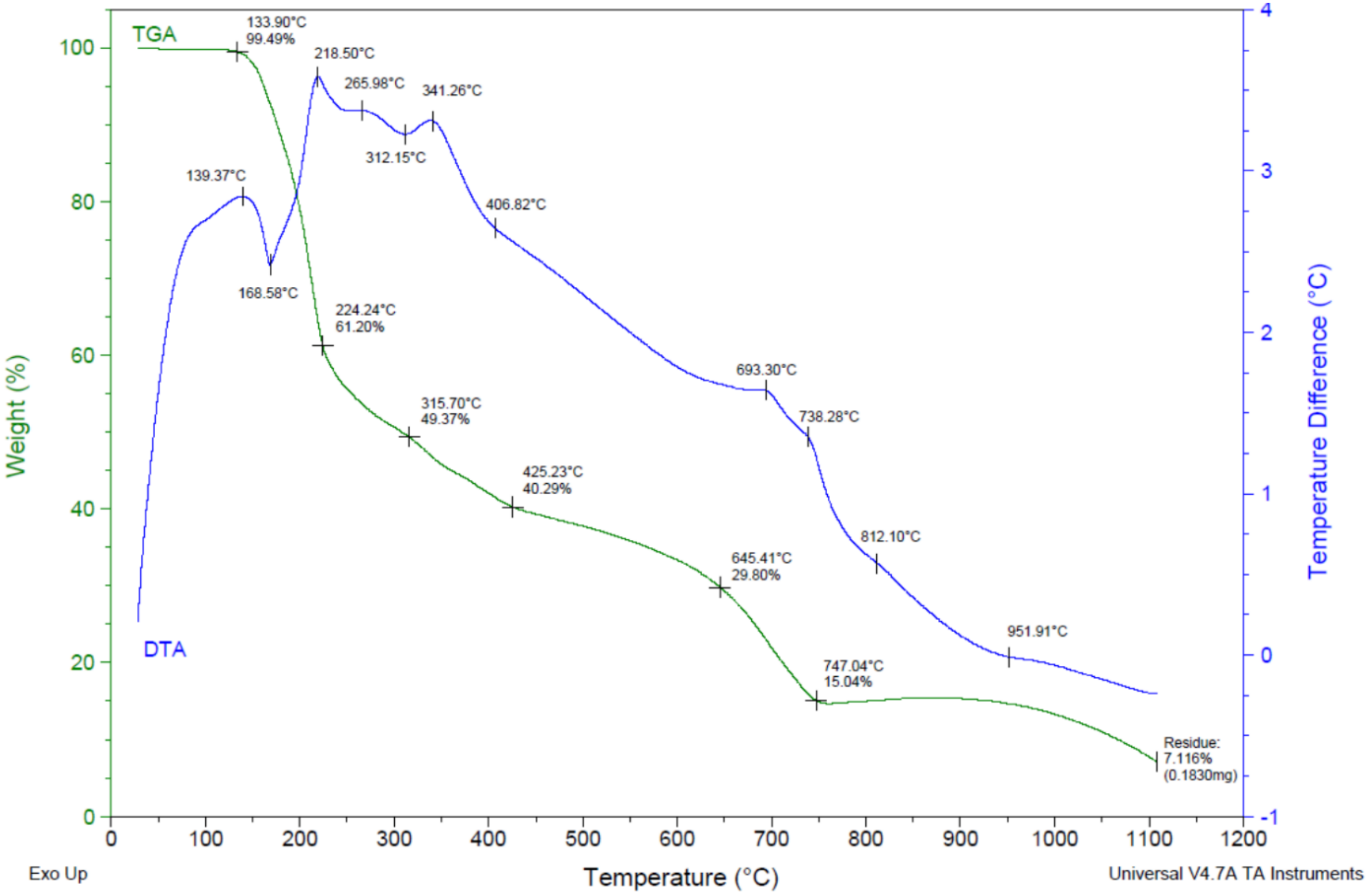

Fig. S3 Thermal Gravimetric Analysis (green) and Difference Temperature Analysis (blue) of compound (I)
